# Allelic expression imbalance of *PIK3CA* mutations is frequent in breast cancer and prognostically significant

**DOI:** 10.1038/s41523-022-00435-9

**Published:** 2022-06-08

**Authors:** Lizelle Correia, Ramiro Magno, Joana M. Xavier, Bernardo P. de Almeida, Isabel Duarte, Filipa Esteves, Marinella Ghezzo, Matthew Eldridge, Chong Sun, Astrid Bosma, Lorenza Mittempergher, Ana Marreiros, Rene Bernards, Carlos Caldas, Suet-Feung Chin, Ana-Teresa Maia

**Affiliations:** 1 Faculty of Medicine and Biomedical Sciences (FMCB), Universidade do Algarve, Faro, Portugal; 2 Center for Research in Health Technologies and Information Systems (CINTESIS), Universidade do Algarve, Faro, Portugal; 3 ProRegeM-PhD Program in Mechanisms of Disease and Regenerative Medicine, Universidade do Algarve, Faro, Portugal; 4 Cancer Research UK Cambridge Institute, Li Ka Shing Centre, University of Cambridge, Robinson Way, Cambridge, UK; 5 Division of Molecular Carcinogenesis, The Netherlands Cancer Institute, Amsterdam, The Netherlands; 6 Department of Oncology, University of Cambridge, Cambridge, UK; 7 Cancer Research UK Cambridge Cancer Centre, Cambridge, UK; 8 Present Address: The Research Institute of Molecular Pathology, Vienna, Austria; 9 Present Address: DKFZ, Heidelberg, Germany

**Keywords:** Breast cancer, Translational research

## Abstract

*PIK3CA* mutations are the most common in breast cancer, particularly in the estrogen receptor-positive cohort, but the benefit of PI3K inhibitors has had limited success compared with approaches targeting other less common mutations. We found a frequent allelic expression imbalance between the missense mutant and wild-type *PIK3CA* alleles in breast tumors from the METABRIC (70.2%) and the TCGA (60.1%) projects. When considering the mechanisms controlling allelic expression, 27.7% and 11.8% of tumors showed imbalance due to regulatory variants in cis, in the two studies respectively. Furthermore, preferential expression of the mutant allele due to cis-regulatory variation is associated with poor prognosis in the METABRIC tumors (*P* = 0.031). Interestingly, ER−, PR−, and HER2+ tumors showed significant preferential expression of the mutated allele in both datasets. Our work provides compelling evidence to support the clinical utility of *PIK3CA* allelic expression in breast cancer in identifying patients of poorer prognosis, and those with low expression of the mutated allele, who will unlikely benefit from PI3K inhibitors. Furthermore, our work proposes a model of differential regulation of a critical cancer-promoting gene in breast cancer.

## Introduction

Activating oncogenic mutations are often characterized by gain-of-function single-base alterations or focal DNA copy-number amplification, where the gain of just a single copy of a mutant allele is sufficient for tumorigenesis^1^. These gains change the stoichiometric balance between mutant and wild-type alleles and are selected for in cancers, affecting approximately half of all oncogenic driver mutations^1^. Ultimately, they could dictate prognosis and therapeutic sensitivity.

However, the impact of gene dosage differences of oncogenic mutations generated at the gene expression level has been largely unexplored. Genetic variation and mutations regulate gene expression in an allele-specific manner—known as cis-regulatory variation^2^—by altering protein and miRNA binding, for example. Normal cis-regulatory variation affects most of the human genome in all tissues and generates the wealth of phenotypic variation seen in species^3–5^. Moreover, an extensive contribution from noncoding variants to RNA alterations was recently observed in tumors^6^, including allelic imbalance of somatic mutations^7^.

Nevertheless, one unsolved aspect is how much each mechanism contributes to generating allelic imbalances in expression and whether they do it independently or in synergy. In breast tissue, germline regulatory variation is associated with disease risk^8^ and affects frequently mutated genes^9^. We and others have shown that variants affecting the expression levels of *BRCA1* and *BRCA2* modify the risk of breast cancer in germline mutation carriers^10,11^. We found that carriers of germline nonsense mutations in the tumor suppressor gene *BRCA2* were at a lower risk of developing breast cancer when the remaining wild-type allele was highly expressed^11^.

Here, we hypothesize that cis-regulatory variation also modulates the penetrance of oncogenic coding mutations. In the context of a gene cis-regulated by a genetic variant generating imbalanced allelic expression, we postulate that an oncogenic activating mutation in the same gene will have a different clinical impact depending upon whether it occurs in the preferentially expressed allele or the less expressed one. We tested this model in the context of heterozygous mutations in *PIK3CA*, the most frequently mutated gene in breast cancer. First, we investigated whether normal cis-regulatory variation regulated the expression of *PIK3CA* in normal breast tissue. Then, we calculated allelic expression ratios between mutant and wild-type copies in tumors from two large breast cancer datasets—METABRIC and TCGA—both normalized for DNA copy number or not. Finally, we correlated the allelic expression ratios with clinical data. This approach allows us to distinguish between expression imbalances generated from cis-regulatory variation alone, altered DNA copy number, or both mechanisms.

## results

### Normal cis-regulatory variation affects *PIK3CA* expression in healthy breast tissue

**Fig. 1:**
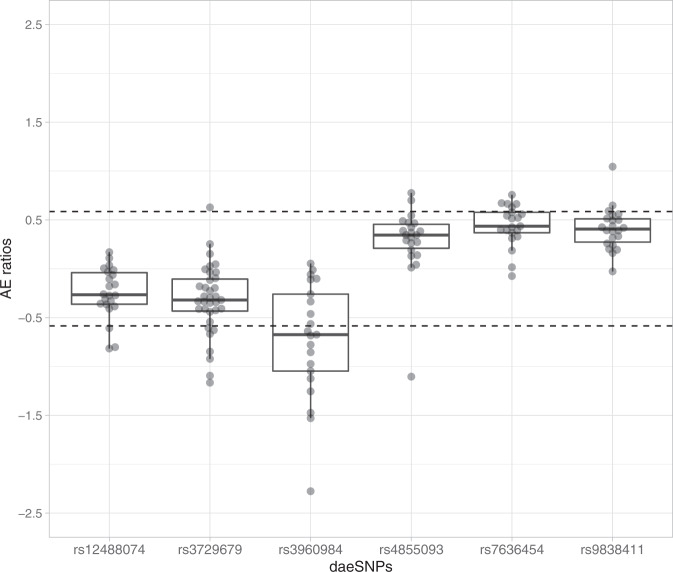
Cis-regulatory variation impacts on *PIK3CA* gene expression in normal breast tissue. AE ratios for six daeSNPs in the *PIK3CA* gene region, each dot is a heterozygous individual for the corresponding variant indicated in the x-axis, dotted lines delimit the levels of 1.5-fold difference for either allele preferential expression ($\left|{{{\rm{AE}}}}\right|=0.58$). Boxplots display the median, the lower and upper hinges corresponding to the first and third quartiles, and lower and upper whiskers corresponding to the smallest and largest values from the 1.5 * IQR (interquartile range), respectively.

To investigate whether cis-regulatory variation modulates the expression of *PIK3CA* in normal breast tissue, we analyzed data from previous allelic expression analysis of normal breast tissue from 64 healthy donors^12^. We calculated the ratio of expression of one allele by the other in heterozygous variant positions, which is a robust approach to detect cis-acting variant effects, as it cancels out the trans effects that act on the same gene and influence both alleles equally. We found six variants in *PIK3CA* displaying differential allelic expression (daeSNPs) (see “Methods”) (Fig. 1). Of these six, only rs3729679 is not in strong linkage disequilibrium (LD) with the others (Supplementary Table 1). rs3960984 showed the largest proportion of heterozygotes displaying allelic differences (57%), while three other daeSNPs shared the smallest fraction (14%): rs12488074, rs4855093, and rs9838411.

In the daeSNPs rs7636454, rs3960984, rs12488074, and rs9838411, the ratios showed a unilateral distribution, with samples displaying preferential expression towards the same allele. These patterns of allelic expression ratios’ distribution suggest that the daeSNPs at which allelic expression is being measured and the possible functional regulatory variants (rSNPs) are in strong, yet incomplete, LD with each other^13^.

While the mapping analysis carried out to identify candidate rSNPs did not find a significant association after multiple testing correction (Supplementary Table 2), one of the variants with nominal *P* value ≤ 0.05, rs2699887 (Wilcoxon two-sample test estimated difference of 0.22, 95% CI = [0.031-Inf]) (Supplementary Fig. 1A), showed great regulatory potential. Namely, it is an eQTL (expression quantitative trait locus) for *PIK3CA* (*P* = 0.011, Supplementary Fig. 1B) in tumors from METABRIC^14^, is located at its promotor region and at a DNAse I hypersensitivity site (Supplementary Fig. 1C), and is bound by POL2 in a breast cancer cell line (Supplementary Table 3). In-silico functional analysis of this variant suggested a disruption of the binding motif of the transcription factor NF-YA (Supplementary Fig. 1D), and in vitro studies revealed a preferential protein::DNA binding to the minor T allele of rs2699887, which is associated with higher expression of *PIK3CA* in tumors (Supplementary Fig. 1E).

### Preferential expression of the *PIK3CA* mutated alleles is frequent in breast tumors

**Fig. 2:**
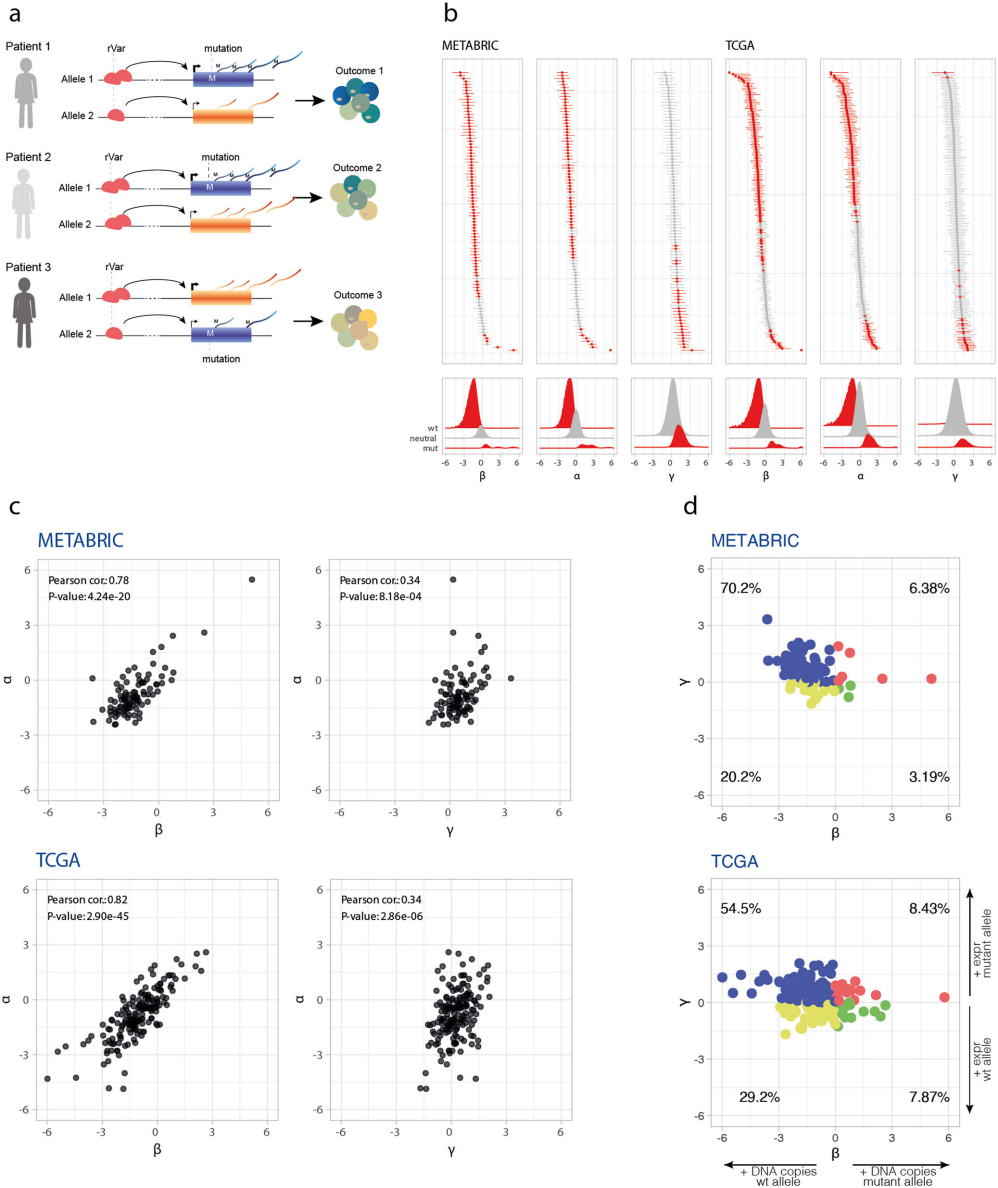
Mutant allelic imbalance in gene expression of somatic missense *PIK3CA* mutations is frequent in breast tumors, particularly for preferential expression of the mutant allele. **a** Schematic representation of the hypothesis: cis-acting regulatory variants (rVar), either from germline or somatically acquired, generate different relative allelic expression ratios of mutant and wild-type alleles, resulting in tumors of different prognosis. **b** Top: log ratio *α*, *β*, and *γ* 89% credible intervals (CI) in breast tumors. Bottom: CIs collective posterior distribution split according to imbalance. A sample is deemed imbalanced if the CI does not cross zero. Samples with significant imbalance are displayed in red. **c** Correlation analysis of *α* vs. *β* and *α* vs. *γ*, showing that both genomic copy-number dosage and allelic expression regulation contribute to imbalances in the expression of mutated alleles in tumors. Point coordinates are Maximum A Posteriori probability estimates (MAP) of the 89% CIs. **d** Comparison of matched *γ* and *β* values, showing predominance of tumors with a preferential allelic expression of the mutated allele. Point coordinates are Maximum A Posteriori probability estimates (MAP) of the 89% CIs.

Changes in DNA copy number in tumors are associated with changes in gene expression in cis^1,14–17^ leading to dosage imbalances of coding mutations^7^. However, these differences can also be due to germline and somatic cis-regulatory variation, but their effect on mutation dosage imbalance is underexplored. So, we set out to assess whether *PIK3CA* somatic mutations would have their functional effects, or penetrance, modified by imbalances in allelic expression generated by regulatory variants. We hypothesized that preferential expression of a gain-of-function mutation would have a more substantial clinical impact than those occurring in lowly expressed alleles, thus generating intertumor clinical heterogeneity (Fig. 2a). To test this, we carried mutant vs. wild-type allelic expression analysis in breast tumor samples carrying somatic *PIK3CA* missense mutations on two independent sets of data, the METABRIC (*n* = 94) and the TCGA (*n* = 178) projects. Supplementary Table 4 presents a summary description of the two datasets and Supplementary Fig. 2 shows the number, location, and amino acid alterations of the mutations across the two datasets.

Next, we calculated three allelic ratios from DNA-seq and RNA-seq data for each mutation:

1. $\alpha ={\log }_{2}$ (number of mutant RNA-seq reads/number of wild-type RNA-seq reads), i.e., the net mutant allele expression imbalance;
2. $\beta ={\log }_{2}$ (number of mutant DNA-seq reads/number of wild-type DNA-seq reads), i.e., the mutant allele relative copy-number;
3. *γ* = *α* − *β*, i.e., the net mutant allele expression imbalance normalized for the DNA allelic copy-number imbalances, which corresponds to a putative mutant allele expression imbalance due to cis-regulation.

In this way, *α* reports on the net allelic expression imbalance, generated by different mechanisms including copy-number aberrations, cellularity differences, and cis-regulatory variation, while *γ* reports specifically on the contribution from cis-regulatory variation (rVar in Fig. 2a), including normal genetic variation, somatic noncoding mutations, and allelic epigenetic changes. Figure 2b displays the distributions of the different ratios.

We found that net mutant allele expression imbalances (*α* ratio) are frequent in breast tumors, at 70.2% in METABRIC (66 out of 94) and 60.1% in TCGA (107 out of 178). The same is true for *γ* ratios, at 27.7% for METABRIC (26 out of 94) and 11.8% for TCGA (21 out of 178), indicating that cis-regulatory effects acting on mutations are also frequent in breast tumors. In both sets, we found samples with striking net preferential allelic expression for the mutant allele (maximum 44.8-fold and 220-fold in METABRIC and TCGA, respectively), but not so for the preferential expression of the wild-type allele (fold differences of 5.4 and 29 in METABRIC and TCGA, respectively) (Fig. 2b). Similarly, the mutant allele’s most pronounced preferential expression trend was found for the *γ* ratio, 10- and 4.2-fold for METABRIC and TCGA, respectively, albeit with smaller fold differences between alleles.

Interestingly, we observed that within the samples with significant mutant allele expression imbalance due to cis-regulatory variation there was a significant prevalence of samples that preferentially expressed the mutated allele in both datasets (binomial test Prob. = 1, 89%−CI = [0.89, 1.00], *P* = 3 × 10^−8^ for METABRIC and Prob. = 0.90, 89%−CI = [0.73, 0.98], *P* = 2 × 10^−4^ for TCGA).

### Cis-regulatory variants contribute significantly to imbalances in the expression of mutant alleles

Next, hypothesizing that both copy number and cis-regulatory variants are the major contributors to allelic expression, we set out to assess the contribution of each mechanism toward the net mutant allele expression imbalances detected in these tumors. First, we found positive correlations between net allelic expression and both copy number and cis-regulatory variation (Fig. 2c), albeit with an effect for the copy number over the double the size of that found for cis-regulatory variation (average Pearson correlation *r*^2^ = 0.80 and 0.34, respectively). Next, we considered the variance (Var) of the net allelic expression as the sum of the effects of both mechanisms, plus the covariance (Cov) accounting for predicted non-mutual exclusion of mechanisms acting on any given allele:

1 $${{{\rm{Var}}}}(\alpha )={{{\rm{Var}}}}(\beta )+{{{\rm{Var}}}}(\gamma )+2\,{{{\rm{Cov}}}}(\beta ,\gamma ),$$

we calculated the contribution of cis-regulatory variation to the variance of net allelic expression as $\left({{{\rm{Var}}}}(\gamma )+{{{\rm{Cov}}}}(\beta ,\gamma )\right)/{{{\rm{Var}}}}(\alpha )$. Here, we found that cis-regulatory variants explain 20.6% and 14.4% of the variability of net mutant allelic expression seen in METABRIC and TCGA, respectively (Supplementary Table 5).

Finally, assessing how the two mechanisms act simultaneously on each tumor, we found that the majority of samples (70.2% and 54.5% for the METABRIC and TCGA, respectively) had positive *γ* and negative *β* values (Fig. 2d), suggesting that although the mutant allele was in lower genomic quantity, it was nevertheless preferentially expressed compared to the wild-type allele. Interestingly, there were 10.6% and 11.2% samples with positive *α* and negative *β* values, in METABRIC and TCGA respectively. This shows that these tumors overexpress the mutant allele despite this allele being in lower copy number.

Only a minor fraction of samples displayed co-occurring preferential allelic expression and a higher allele copy number of the mutant allele (6.38% and 8.43% for the METABRIC and TCGA, respectively). These results were independent of the effect of tumor cellularity (Supplementary Fig. 3).

### Preferential expression of mutant alleles by cis-regulatory variation associates with poor prognosis

**Fig. 3:**
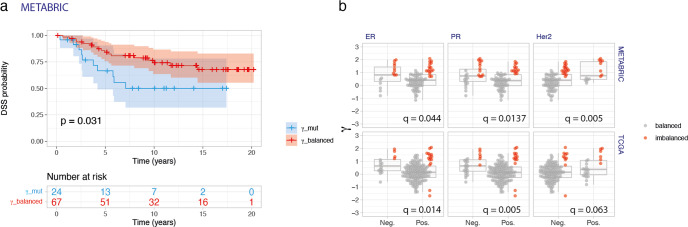
Allelic preferential expression of *PIK3CA* mutations is associated with survival and clinicopathological parameters in breast cancer. **a** Kaplan–Meier curve of disease-specific survival showing the worse prognosis of patients with differential expression of the *PIK3CA* mutations (*γ*_mut_ group, shown in blue) compared to those expressing equimolar levels of mutation and wild-type alleles (*γ*_balanced_ group, shown in red), in METABRIC. Shown below the graph are the numbers of patients at risk per group throughout time. **b** Preferential expression of the mutated allele is associated with ER-negative, PR-negative, and Her2-positive breast tumors. In all graphs, samples were colored according to the significance of the allelic expression imbalance. *q* values indicated correspond to the Wilcoxon rank-sum test with continuity correction, corrected for multiple testing using the Benjamini & Hochberg method. Survival plots indicate the 95%CI as colored shades. Boxplots display the median, the lower and upper hinges corresponding to the first and third quartiles, and lower and upper whiskers corresponding to the smallest and largest values from the 1.5 * IQR (interquartile range), respectively.

To investigate the impact of differential cis-regulation of *PIK3CA*’s mutations on clinical outcome (overall and disease-specific survival), we performed univariate survival analysis with *γ* ratios categorized in three groups, based on the existence of imbalance and its direction, i.e. whether there was significant predominance of expression of the mutated allele *γ*_mut_, of the wild-type allele *γ*_wt_, or balanced allelic expression *γ*_balanced_. We uncovered that the group *γ*_mut_ had a poorer disease-specific survival rate (*P* = 0.031, Fig. 3a) than the *γ*_balanced_ group for METABRIC. The median overall survival for the *γ*_mut_ group was 5.88 years and for the *γ*_balanced_ group was 12.46 years (Supplementary Fig. 4B), whereas, in the disease-specific analysis, the mean survival of the *γ*_mut_ patients was 7.07 years, and 41% of patients died during the length of the follow-up, in comparison with 25.3% deaths in the *γ*_balanced_ group (Fig. 3a).

The categorized *γ* ratios were not significantly associated with overall survival in the multivariate analysis (Supplementary Fig. 5). However, some of the variables that are usually independent prognosis factors, such as PR and HER2 statuses, were not significantly associated with survival either in this analysis. In the TCGA set, there was a trend toward a worse disease-specific survival of those patients whose tumors preferentially express the mutated allele (Supplementary Fig. 6). However, due to the relatively shorter follow-up time of this dataset (median ~1 year) and the fact that tumors were mainly Luminal A (~61.2% of samples)^18^, the power to detect significant differences is smaller than that of METABRIC. Nevertheless, the joint analysis of the two datasets showed a significantly worse disease-specific survival of the *α*_mut_ group of patients, with a concordant trend in the *γ*_mut_ group (Supplementary Fig. 7).

### *PIK3CA* preferential mutant allele expression associates with clinicopathological variables

Next, we sought to investigate whether *PIK3CA*’s differential mutant allele was associated with known prognostic clinicopathological variables, namely hormone receptors (ER, PR) and HER2 amplification, which are directly and indirectly connected to gene expression regulation, respectively.

For both datasets, we observed that preferential mutant allele expression driven by cis-regulatory variation (*γ*) was associated with markers of worse prognosis, namely it was significantly higher in ER-negative tumors and PR-negative tumors, and in HER2-positive tumors only in METABRIC (Fig. 3b). When evaluating the contribution of cis-regulatory variation to this association, we also found that higher average *γ* values associated with lower PR expression (*P* = 0.040) and HER2-positive tumors (*P* = 0.025), but we did not find a significant association with ER expression (*P* = 0.129) (Supplementary Fig. 8).

Given these results, we took *γ* into consideration in the survival analysis within the expression subgroups of ER, PR, and HER2, but did not find significant differences in overall and disease-specific survival in METABRIC (Supplementary Fig. 4).

Considering other known prognostic variables, including tumor size, grade, and molecular subtypes (PAM50^19^ and IntClust^14^, we found a significant association between *γ* ratios and PAM50 subtypes only in METABRIC (*q* = 0.027) (Supplementary Table 6 and Supplementary Fig. 9).

Finally, we did not find an association between the candidate germline regulatory variant rs2699887 and *γ* or clinical outcome, suggesting germline variants are unlikely to be involved in the significant associations described above (data not shown).

However, supporting the involvement of somatic cis-regulatory variants instead, we found smaller fold changes and less samples with imbalances measured at common *PIK3CA* variants in normal-matched tissue data than those measured at mutations in tumor tissue (Supplementary Fig. 10).

## Discussion

Our work reveals the role of cis-regulatory variation acting on *PIK3CA* somatic mutations as modifiers of mutation penetrance. We show for the first time that allelic expression imbalance between *PIK3CA*’s mutant and wild-type alleles is common and prognostic in breast cancer.

Particularly, preferential expression of the mutant allele is significantly more common than that of the wild-type allele, and considering that *PIK3CA* is an oncogene, one possibility is that positive selection could have a role in generating this difference, which should be further investigated. Furthermore, we also found that allelic imbalance in expression observed for the mutant alleles in the tumors was greater than that observed for single-nucleotide polymorphisms in the normal-matched tissue of patients. These findings support the hypothesis of somatic regulatory mutations involvement in generating the imbalances observed in the tumors. While genomic allelic imbalance remains the largest determinant of allelic expression dosage (showing the highest correlation with and contributing the most to the variability observed in net allelic expression), cis-regulatory variation is also significantly correlated with net allelic expression and explains ~16% of its variability across samples in these sets of tumors.

The analysis of RNA-seq data from two independent cohorts of tumor samples, the METABRIC and TCGA projects, strongly supports our findings.

Moreover, we show that preferential expression of the mutant allele due to cis-variation is associated with poor prognosis variables, such as ER-negative, PR-negative, and Her2-positive statuses^20,21^. In the METABRIC dataset, we also found that preferential expression of the mutant allele was associated with worse overall and disease-specific survival. The high stringency in calling imbalance and the focus on a specific type of mutation in one gene, limits this study in terms of the sample size analyzed, but on the other hand it provides the simplest scenario for testing our hypothesis. Interestingly, the joint analysis of the datasets revealed some level of association between disease-specific survival and the preferential expression of the mutant allele, both net and due to cis-regulation, reaffirming the clinical importance of the expression level of a mutation commonly associated with aggressive tumors. In addition, some tumors presented preferential expression of the wild-type allele of *PIK3CA*, suggesting that these mutations are lowly expressed and possibly passenger events.

Besides the potential use of our findings as a prognosis biomarker in the clinic, these results may also have therapeutic implications. Some of the major clinical challenges in cancer treatment are identifying biomarkers of prognosis and defining which patients will benefit from a given therapy. Particularly, it is crucial to identify patients unlikely to respond to specific therapies to prevent unnecessary drug cytotoxicity without any therapeutic benefits. Our results reveal the importance of considering allelic expression in somatic mutation screens in these two aspects of patient management. Despite the high frequency of *PIK3CA* mutations in breast cancers, the response to PI3K inhibitor therapy has been more challenging than expected, and the prognostic significance of detecting somatic *PIK3CA* mutations in breast tumors is unclear^22^. Relevant to this discussion, we have previously shown that the presence of *PIK3CA* mutations confer a poorer prognosis in patients with ER-positive breast cancer only when stratified into copy-number driven subgroups (IntClust 1+, 2+, 9+)^23^.

In this study, we provide new evidence for the prognostic significance of these mutations at the expression level in breast tumors. Particularly for tumors with significant preferential expression of the wild-type allele, this prognostic significance has a potential impact on therapy response and clinical management since one may hypothesize that little to no benefit would come from treatment in the cases not expressing the targetable mutation.

Further studies evaluating the allelic expression of mutant oncogenes in the tumors of patients enrolled in molecular-driven trials will clarify this impact.

More challenging is determining which cis-regulatory mechanisms are promoting allelic expression imbalances. Both inherited^11,24^ and acquired variants^6,25–27^ can affect gene expression in an allelic manner^28,29^.

Here we show that normal cis-regulatory variation regulates *PIK3CA*’s expression in normal breast tissue, with the possible contribution of rs2699887 as a regulatory variant. We also found that the heterozygotes for “rs2699887” were associated with higher expression of the *PIK3CA* gene compared to the common homozygotes. Although there is published data supporting the clinical association of rs2699887 with poor prognosis in other cancers^30,31^, linked to an increase in PI3K signaling, there is still some data supporting the opposite association^32,33^. We did not find an association between rs2699887 and survival, which opens the possibility for other mechanisms besides normal cis-regulatory variation to be considered as contributors to the preferential allelic expression in these tumors (data not shown).

Double *PIK3CA* mutations in the same allele are frequent in breast tumors^34^, and the impact of noncoding mutations in cancer is just starting to be explored^6^. So, a possibility is that the combination of noncoding and coding mutations in the same gene might be underlying the allelic expression imbalances we are detecting.

Further studies on allelic expression imbalances of activating mutations, and even inactivating ones, should further reveal the contribution of cis-regulatory mechanisms in tumor development and progression. Particularly interesting to determine is whether the coding mutation originates in an allele predisposed with higher expression, or whether a sequence of somatic events introduces the coding activating mutation and additional cis-regulatory noncoding mutations. The answers could have significant repercussions on our understanding of tumor evolution.

In summary, we show that differential expression between the mutant and wild-type alleles of *PIK3CA* is common in breast cancer and with a significant contribution from allele-specific cis-regulatory effects. We further show that mutant allele differential expression is associated with clinical parameters such as ER, PR, and HER2 statuses and is prognostically significant.

Collectively, our work establishes the prognostic relevance of allele-specific transcriptional regulation of *PIK3CA* somatic mutations. It also supports a shift in the mutation testing in patient management, where the level of expression of these mutations should be considered, besides the detection at the DNA level.

## Methods

### Subjects

Normal breast and tumor samples were obtained with the written informed consent from donors and appropriate approval from local ethical committees, with the detailed information described in the respective original publications: normal tissue^9^, METABRIC^14^, TCGA^35^.

### Differential allelic expression analysis

DNA and total RNA from 64 samples of normal breast tissue were hybridized onto Illumina Exon510S-Duo arrays (humanexon510s-duo), and data were analyzed as described before^12^. In short, after sample filtering and normalization, variants with average RNA log2 allelic intensity values greater than 9.5 and heterozygous in five or more samples were kept for further analysis.

Allelic log ratios were calculated for RNA and DNA intensity data:

2 $$\log \, {{{\rm{ratio}}}}={\log }_{2}(A)-{\log }_{2}(B),$$

for alleles A and B.Next, variants that showed significant differences between the RNA log ratios between heterozygous (AB) and homozygous groups (AA and BB) (two-sample Student’s *t* test, *P* value < 0.05) were selected for differential allelic expression analysis.

Allelic expression (AE) ratios were normalized for allelic DNA content:

3 $${{{\rm{AE}}}}\,{{{\rm{ratio}}}}={{{\rm{RNA}}}}\,\log\, {\hbox{-}}\, {{{\rm{ratio}}}}\,-\,{{{\rm{DNA}}}}\,\log \,{\hbox{-}}\, {{{\rm{ratio}}}}$$

Differential allelic expression (DAE) at the sample level was defined as ∣AE ratio∣ ≥ 0.58 (1.5-fold or greater between alleles), based on previous studies using microarray data^3,36^. Variants with at least 10% and three heterozygous samples displaying DAE were further classified as daeSNPs.

Linkage disequilibrium (LD) between daeSNPs was evaluated using the genetic variant-centered annotation browser SNiPA^37^.

### Genotype imputation analysis on normal breast tissue samples

Illumina Exon 510 Duo germline genotype data from the 64 samples that passed microarrays quality control, were filtered to keep variants with call rates ≥85%, minor allele frequency >0.01, and Hardy–Weinberg equilibrium with *P* > 1 × 10^−5^. Next, genotypes were imputed with MACH1.0^38^ for all additional known variants on chromosome 3, using as reference panel the phased CEU panel haplotypes from the HapMap3 release (HapMap3 NCBI Build^39^, CEU panel —Utah residents with Northern and Western European ancestry), and the recommended two-step imputation process: model parameters (crossover and error rates) were estimated before imputation using all haplotypes from the study subjects and running 100 Hidden Markov Model (HMM) iterations; then genotypes were imputed using the model parameter estimates from the previous round. Imputation results were filtered based on an ${{{\rm{rq}}}}\,{{{\rm{score}}}}\ge 0.3$^38^, a platform-specific measurement of variant imputation uncertainty.

### Differential allelic expression (DAE) mapping analysis on normal breast tissue samples

Differential allelic expression mapping analysis was performed by stratifying AE ratios at each *PIK3CA* daeSNP according to the genotype at variants located within ±250 Kb.

A Mann–Whitney test was applied to test if the mean of the absolute AE ratios of the heterozygous samples was greater than those of the combined reference and alternative allele homozygous samples. Correction for multiple testing was performed using BH method (p.adjust, R stats 4.0.3 package^40^) and limiting the significance to *q* values ≤0.05.

### Functional annotation of DAE mapping associated variants

Variants in LD with SNPs with DAE mapping nominal-p-value ≤0.05 were retrieved using the function get_ld_variants_by_window from the ensemblr R package (https://github.com/ramiromagno/ensemblr) using the 1000 GENOMES project data (phase_3) for the EUR population and an *r*^2^ > 0.95. These proxy SNPs were assessed for overlap with epigenetic marks derived from the Encyclopedia of DNA Elements (ENCODE) and NIH Roadmap Epigenomics projects, such as chromatin states (chromHMM) annotation, regions of DNase I hypersensitivity, transcription factor binding sites, and histone modifications of epigenetic markers (H3K4Me1, H3K4Me3, and H3K27Ac) (http://genome.ucsc.edu/ENCODE/) for normal human mammary epithelial cells (HMECs), human mammary fibroblasts (HMFs), BR.MYO (breast myoepithelial cells) and BR.H35 (breast vHMEC) and two breast cancer cell lines MCF-7 and T47D. We prioritized variants located on either active promoter or enhancer regions in mammary cell lines, and for which ChIP-Seq data indicated protein binding or position weight matrix (PWM) scores predicted differential protein binding for different alleles. Two publicly available tools, RegulomeDB and HaploReg v4.1, and the MotifBreakR Bioconductor package, were also used to evaluate those candidate functional variants^39,41,42^.

### Electrophoretic mobility shift assay (EMSA)

MCF-7 (ER-positive) and HCC1954 (ER-negative) breast cancer cell lines were cultured in DMEM and RPMI culture media, respectively, supplemented with 10% FBS and 1% PS (penicillin and streptomycin). Nuclear protein extracts were prepared using the Thermo Scientific PierceTM NER kit, according to the manufacturer’s instructions. Oligonucleotide sequences corresponding to the C (common) and T (minor) alleles of rs2699887 (5’-AGCGTGAGTAGAGCGCGGA[C/T]TGGCCGGTAGCGGGTGCGGTG-3’) were labeled using the Thermo Scientific Pierce Biotin 3’ End DNA Labelling Kit, according to the manufacturer’s instructions. Oligonucleotides with known binding motifs for NF-YA^43^ and E2F1^44^ were used in competition assays. Undiluted antibodies used for supershift competition assays were NF-YA (H-209) (Santa Cruz Biotechnology, SC-10779X) and HMGA1a/HMGA1b (Abcam, ab4078). EMSA experiments were performed using the Thermo Scientific LightShiftTM Chemiluminescent EMSA Kit, using the buffer and binding reaction conditions previously described^8^. Each EMSA was repeated at least twice for all combinations of cell extract and oligonucleotide, which were also tested in serial dilution amounts.

### Breast tumor samples

The METABRIC dataset of tumor samples included 2433 samples from the METABRIC project^14^ with DNA sequencing data, among which 480 were subjected to a capture-based RNA sequencing study^23^. Sequencing libraries were generated as previously described. In brief, sequencing libraries using total RNA generated from frozen tissues with a TruSeq mRNA Library Preparation Kit using poly-A-enriched RNA (Illumina, San Diego, CA, USA) and enriched with the human kinome DNA capture baits (Agilent Technologies, Santa Clara, CA, USA). Six libraries were pooled for each capture reaction, with 100 ng of each library, and sequenced (paired-end 51bp) on an Illumina HiSeq2000 platform. We selected a subset of samples with DNA and RNA sequencing data and PIK3CA missense mutations for further analysis.

The TCGA dataset comprised 695 samples from TCGA breast cancers^35^, from which we selected a subset of 289 samples with PIK3CA missense mutations for further analysis. Supplementary Table 3 summarizes the demographic features and disease characteristics of the two datasets.

### DNA-seq and RNA-seq variants calling in tumors

#### Alignment and preprocessing

Sequence data (FASTQ) mapped to the reference genome (hg19) were aligned using STAR v2.4.1^45^. A two-pass alignment was carried out: splice junctions detected in the first alignment run are used to guide the final alignment. Duplicates were marked with Picard v1.131 (http://picard.sourceforge.net). Genome Analysis Toolkit (GATK) was used for indel realignment and base quality score recalibration^46^.

#### Variant calling and annotations

SNV and indel variants were called using GATK Haplotype Caller. Hard filters using GATK VariantFiltration were applied to variants^46^. Variants were annotated with Ensembl Variant Effect Predictor (VEP)^47^. Heterozygous genotypes were called from DNA data to avoid RNA editing and other RNA-related variants because true allelic imbalance can lead to heterozygous sites being called homozygous in RNA-based genotype calling.

### Analysis of allelic expression imbalances in tumors

Before the analysis, a set of filtering steps was performed to select samples: (1) presence of missense mutations; (2) and a minimum of 30 reads for RNA-seq and DNA-seq data^48–50^.

Clinical data for METABRIC were updated from the original studies with the latest available records. Clinical data for TCGA were imported from https://portal.gdc.cancer.gov/ on November 26, 2018.

#### Filtering of tumor samples

For both datasets —METABRIC and TCGA—, a set of quality control criteria were applied to filter the DNA-seq and RNA-seq samples, namely:

- Keep only samples containing *PIK3CA* missense mutations;
- Keep samples whose coverage at mutated loci is, all together in both alleles, at least 30 reads for both RNA-seq and DNA-seq data^48–50^.

Clinical data of METABRIC patients were updated from the original studies with the latest available records. The TCGA clinical dataset was obtained from cBioPortal^51,52^ on 28 November 2021 by programmatic access with the R package cgdsr.

#### Allelic expression imbalances in tumor data

Allelic expression imbalances are calculated as follows. For each mutated loci, the pair of read counts (*X*, *Y*), for wild-type (*X*) and mutant (*Y*) alleles, respectively, measured either by DNA-seq or RNAseq, are transformed using the log ratios *β*, *α*, and *γ*, which are defined as follows:

4 $$\beta ={\log }_{2}({Y}_{{{{\rm{DNA}}}}}/{X}_{{{{\rm{DNA}}}}}),$$

the DNA mutant allele ratio, which served to control for sequencing artifacts from heterozygous genotypes and to account for differences in variant frequencies in DNA;

5 $$\alpha ={\log }_{2}({Y}_{{{{\rm{RNA}}}}}/{X}_{{{{\rm{RNA}}}}}),$$

that served as a measure of the net allelic expression imbalance in tumors;

6 $$\gamma =\alpha -\beta,$$

the normalized mutant allele expression ratio, a proxy for the mutant allelic expression imbalance due to cis-regulation alone.

#### Statistical inference of allelic expression imbalances

According to these log-ratio definitions, a positive value indicates an imbalance toward the mutant allele, and a negative value an imbalance favoring the wild-type allele. However, the statistical significance of each log ratio depends on the read coverage of each allele, e.g., low read-coverage values are subject to greater random variation, and hence less reliable log ratios and imbalances estimation. To assign a measuring of uncertainty to our imbalances’ estimates, we assumed that the read counts are well modeled by a Beta-Binomial distribution, and following Bayesian reasoning, we estimated 89% credible intervals (CI) and Maximum A Posteriori probability estimates (MAP) for the log ratios *β*, *α*, and *γ* (reported in Fig. 2).

#### Allelic expression imbalances in normal-matched tissue data

Solid normal breast tissue from breast cancer female patients was obtained from TCGA-BRCA. We selected 112 samples with RNA-Seq data, obtained in bam file format. Sequence data were converted to fastq format (samtools^53^), underwent initial quality control (FastQC^54^), and trimming (Trimmomatic^55^). Following QC, six samples were removed from analysis. The remaining sequence data was mapped to the reference genome (hg38) using STAR aligner (v.2.7.7a^45^). Otherwise, alignment, preprocessing and variant calling was performed as described below. RNA data was filtered to contain only heterozygous variants at the DNA level, circumscribed to PIK3CA’s genomic location. DNA data was accessed from TCGA-BRCA’s microarray raw data for 111 of the 112 initial RNA-Seq samples. Genotypes were obtained using the CRLMM algorithm (‘crlmm’ R Bioconductor package,^56^) and quality controlled for HWE, major allele frequency and 10% missing genotypes (‘SNPassoc’ R package^57^). Genotypes were lifted over from hg38 to hg37 (‘rtracklayer’ R Bioconductor package^58^), harmonized (‘GenotypeHarmonizer’^59^), and imputed (Michigan Imputation Server^60^). Obtained genotypes were quality controlled using PLINK^61^ and lifted over back to hg38. Allelic expression imbalances, equivalent to *α* ratios in tumors, were inferred for heterozygous germline variants as described above.

#### Two-sample tests of imbalance ratios with clinical covariates

Association between allelic expression imbalance ratios and clinical data was achieved by bivariate analysis Wilcoxon rank-sum test with continuity correction or Kruskal–Wallis rank-sum test, as indicated in tables and figures. *P* values were adjusted per study using the Benjamini & Hochberg correction and were considered significant when ≤ 0.05.

#### Correlation analysis

Correlation analysis *α* vs *β* and *α* vs *γ* ratios for both sets of samples were performed using a Pearson’s test. All statistical analysis and data visualization were performed using R.

### Survival analyses

Kaplan–Meier plots and multivariate Cox proportional hazard models were used to examine the association between alpha and gamma allelic expression ratios and survival using the survival package from R^62,63^. Death due to all causes was used as the endpoint, and all alive subjects were censored at the date of the last contact. Kaplan–Meier survival curves were compared using the log-rank test.

For the multivariate analysis, Cox proportional hazard model was used to assess the effect of *γ* on the overall survival. Hazard ratios (HRs) and 95% confidence intervals (CI) were estimated by fitting the Cox model while adjusting for age and tumor characteristics, such as size, Scarff–Bloom–Richardson histological grade, clinical stage and estrogen receptor (ER), progesterone (PR), and human epidermal growth factor 2 (HER2) statuses.

For the bivariate analysis, Wilcoxon rank-sum two-sample tests were used to compare *α* and *γ* between different hormone receptor statuses and *q* ≤ 0.05, calculated using the Benjamini & Hochberg method, were considered statistically significant.

### Reporting summary

Further information on research design is available in the Nature Research Reporting Summary linked to this article.

## Supplementary information

Correia_2021_Supplementary_Figures

Supplementary Tables Revised

Reporting Summary

## Data Availability

Microarray raw data are deposited in the Gene Expression Omnibus under accession number GSE35023. Primary data (BAM files) for DNA-seq are deposited at the European Genome-phenome Archive (EGA) under study accession number EGAS00001001753 and may be downloaded upon request and authorization by the METABRIC Data Access Committee. Primary data (BAM files) for RNAseq are available from the authors upon reasonable request. Primary data (BAM files) for DNA-seq and RNAseq from TCGA are deposited in the database of Genotypes and Phenotypes (dbGaP) under the study accession number phs000178.

## Code availability

The filtered data and code for the analysis of mutant allele expression imbalances and the survival analysis can be publicly accessed at https://github.com/maialab/npjbcPIK3CA.
