# Supplementary material for: Allelic expression imbalance of PIK3CA mutations is frequent in breast cancer and prognostically significant
Source: NPJ Breast Cancer. 2022 Jun 8;8:71. doi: 10.1038/s41523-022-00435-9 (PMC9177727; doi:10.1038/s41523-022-00435-9)
Supplement: Supplementary file 1 — Correia_2021_Supplementary_Figures [file 41523_2022_435_MOESM1_ESM.pdf]

**Allelic expression imbalance of *PIK3CA* mutations is frequent in breast cancer and prognostically significant**
Lizelle Correia et al.

## Supplementary Figures

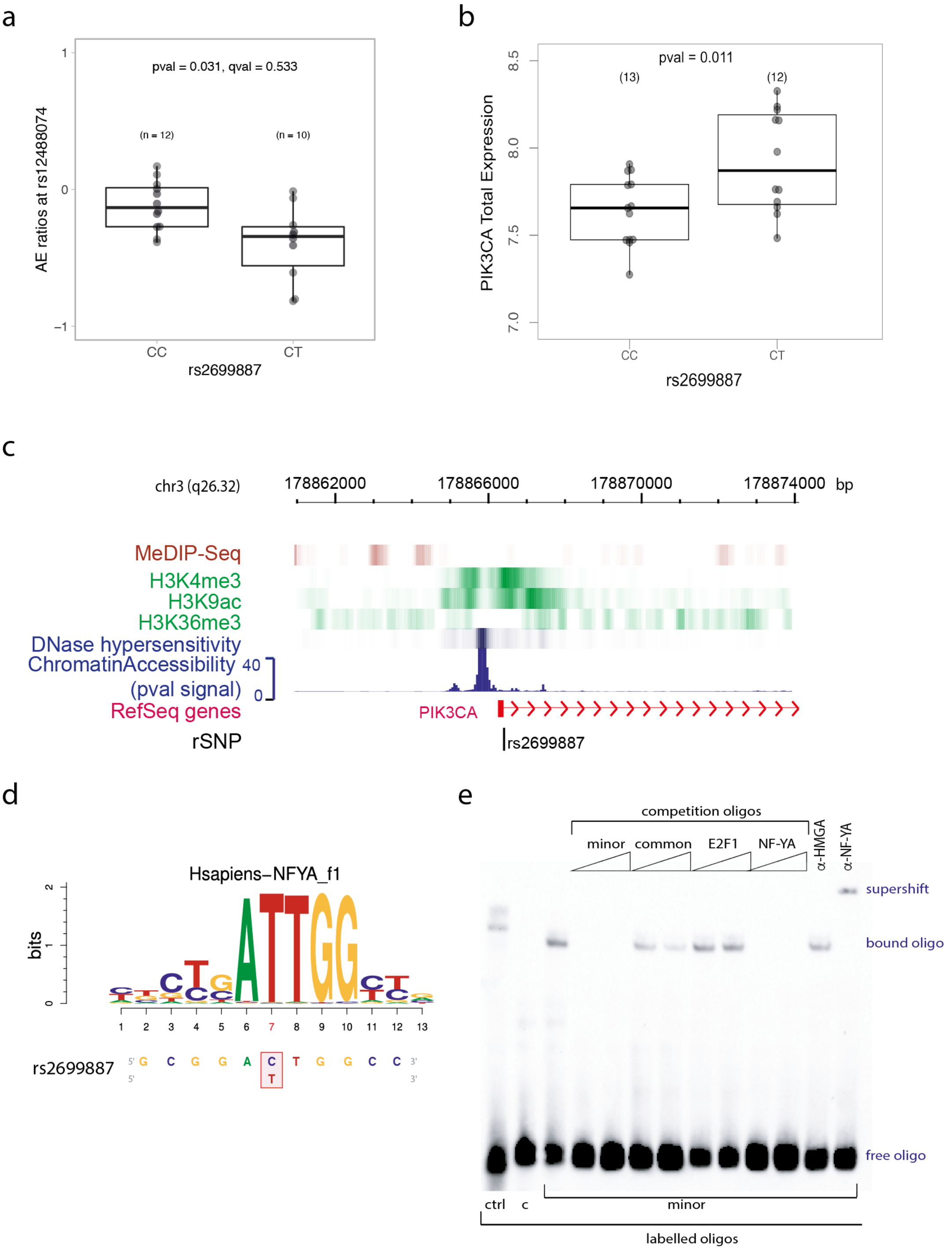


**Supplementary Figure 1 – Functional characterization of *cis*-regulatory variation impacting on *PIK3CA* gene expression in normal breast tissue. a** – Association of genotype at rs2699887 with differential allelic expression of *PIK3CA* measured at rs12488074. Indicated are the p-value of a two-

sided Wilcoxon rank sum test, and the corresponding q-value (multiple test correction with Bonferroni). **b** – rs2699887 is an eQTL for the expression of *PIK3CA* in tumors from METABRIC. P-value indicated corresponds to Student's t-test. **d** – Genomic view of rs2699887's location, showing methylation of surrounding CpG sites (MeDIP-seq), typical active promoter chromatin modification marks (H3K4me3, H3K9ac, H3K36me3), and open chromatin status (*DNAse*I hypersensitivity and Chromatin Accessibility p-value). Data from the Roadmap of Epigenomics project for breast HMECs. **e** – PWM analysis suggested that rs2699887 disrupts an NF-YA binding motif at the four-nucleotide core sequence. **f** – rs2699887 differentially binds protein in vitro. Representative EMSA analysis using biotin-labeled oligonucleotides containing either rs2699887-C allele (indicated as *c* or common) or rs2699887-T allele (indicated as minor), and one positive control (ctrl), using protein extracts of breast cancer cell line HCC1954. Competition assays included one (100x) or two concentrations (10x and 100x), as indicated by the gradient symbol of unlabeled oligonucleotides, which included consensus binding sequences for NF-YA and E2F1. Supershift assays were carried out using monoclonal antibodies against NF-YA and one negative control (antibody against HGMA).

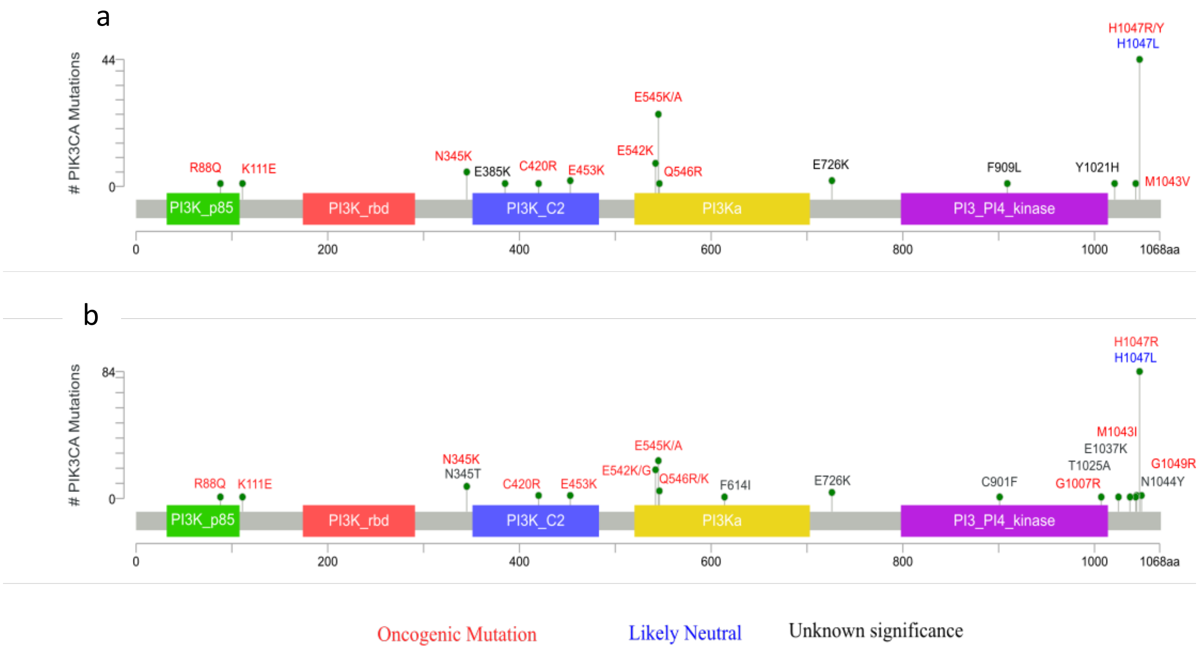


**Supplementary Figure 2** - Genomic distribution and counting of mutations in the *PIK3CA* gene in METABRIC (a) and in TCGA (b) datasets. Green lollipops represent the missense mutations, with the height of the lollipop representing the total number for each mutation. Amino acid changes are indicated in red represent the oncogenic mutations, in blue likely neutral mutations, and in black mutations of unknown significance, according to Ensembl Variant Effect Predictor.

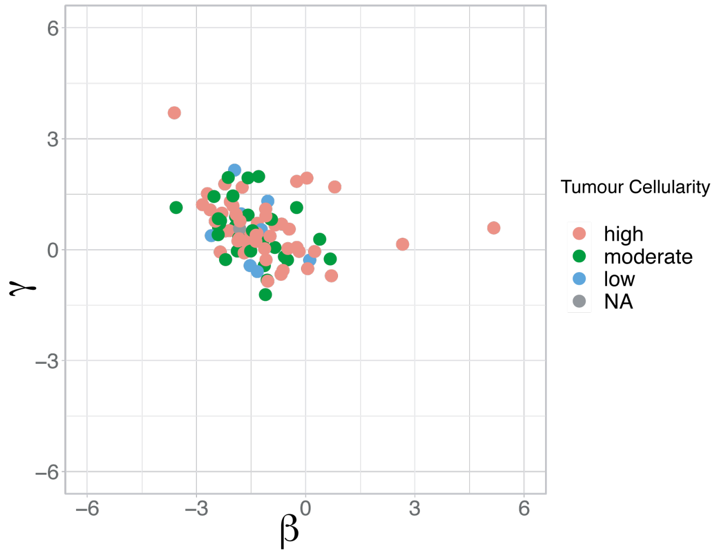


**Supplementary Figure 3 - Comparison of *PIK3CA's* matched β and γ values reveals no pattern of association between ratios and cellularity in METABRIC tumors.** Each dot represents a tumor, and color indicates cellularity levels.

a

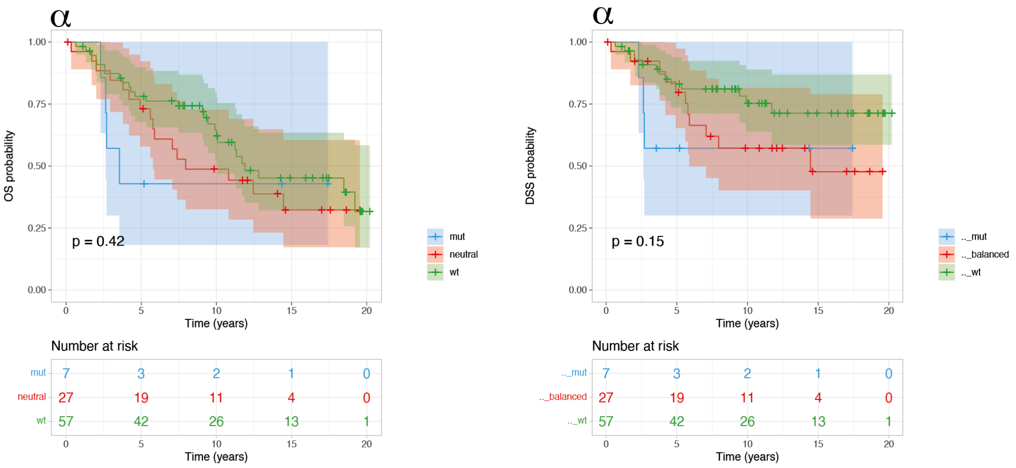


b

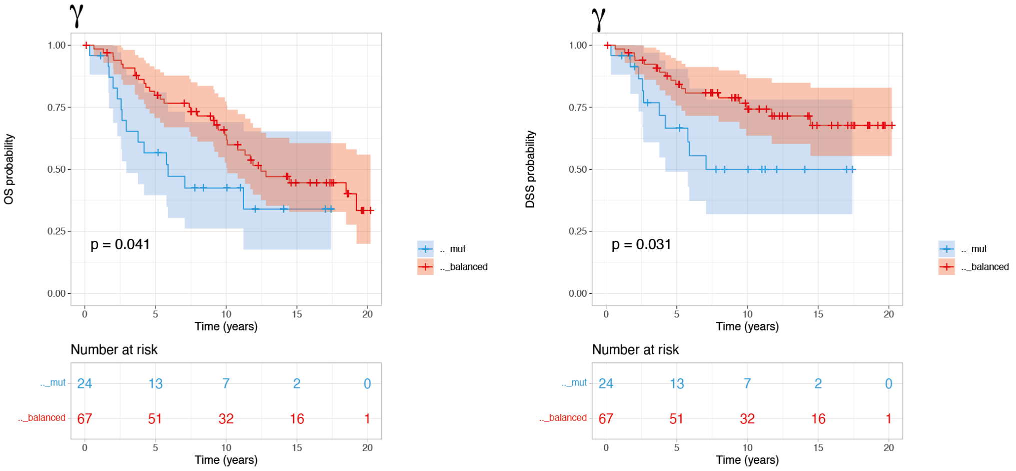


c

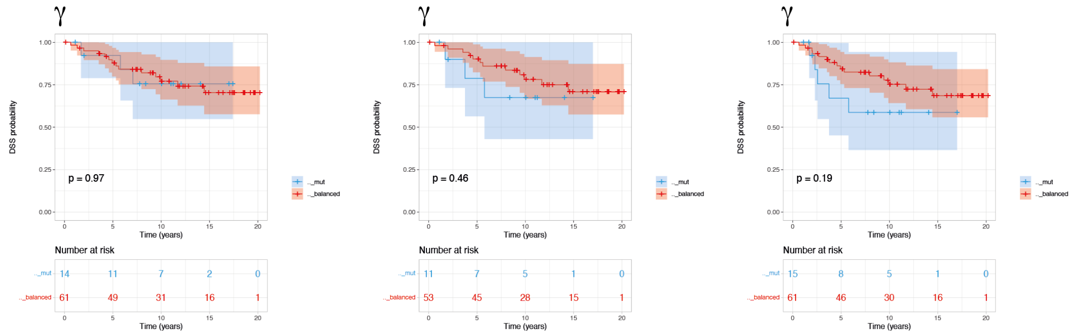


**Supplementary Figure 4 - Survival of mutant allele preferential expression in METABRIC tumors. a** – Kaplan–Meier curves of overall and disease-specific survival of patients stratified for the net differential expression (α) of the *PIK3CA* mutations levels. **b** – Overall and disease-specific survival Kaplan-Meier curves with the patients stratified for the differential expression driven by cis-regulation (γ) of the *PIK3CA* mutations levels, confirming worse survival of patients with tumours preferentially expressing the mutant allele compared to those with equimolar expression of the two alleles. **c** –Kaplan-Meier curves showing no significant association between disease specific survival and the differential expression of the mutations due to cis-regulation in patients grouped by hormone receptor status. ER = estrogen; PR = progesterone; HER2 = human epidermal growth factor 2 receptor. Shown below the graphs are the numbers of patients at risk per group throughout time.

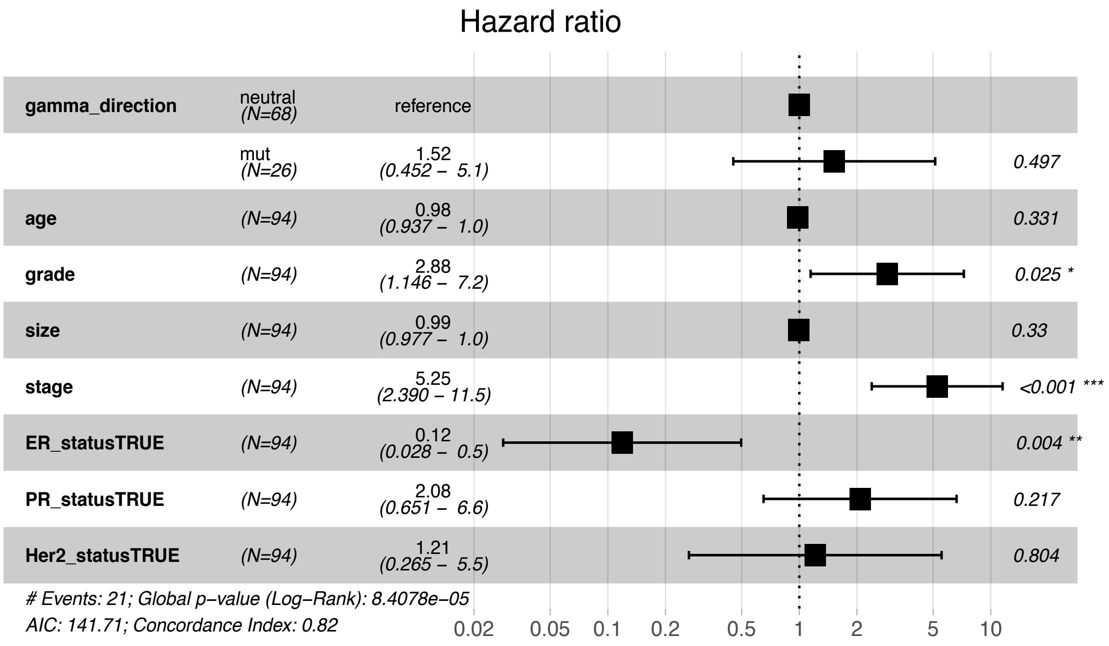


**Supplementary Figure 5 - Survival of mutant allele preferential expression in METABRIC tumors.** Forest plot from multivariate Cox regression model showing the association of PIK3CA's γ ratios and disease specific survival, adjusted for age, grade, ER, PR and HER2 statuses, and tumor stage. (ER = estrogen; PR = progesterone; HER2 = human epidermal growth factor 2 receptor).

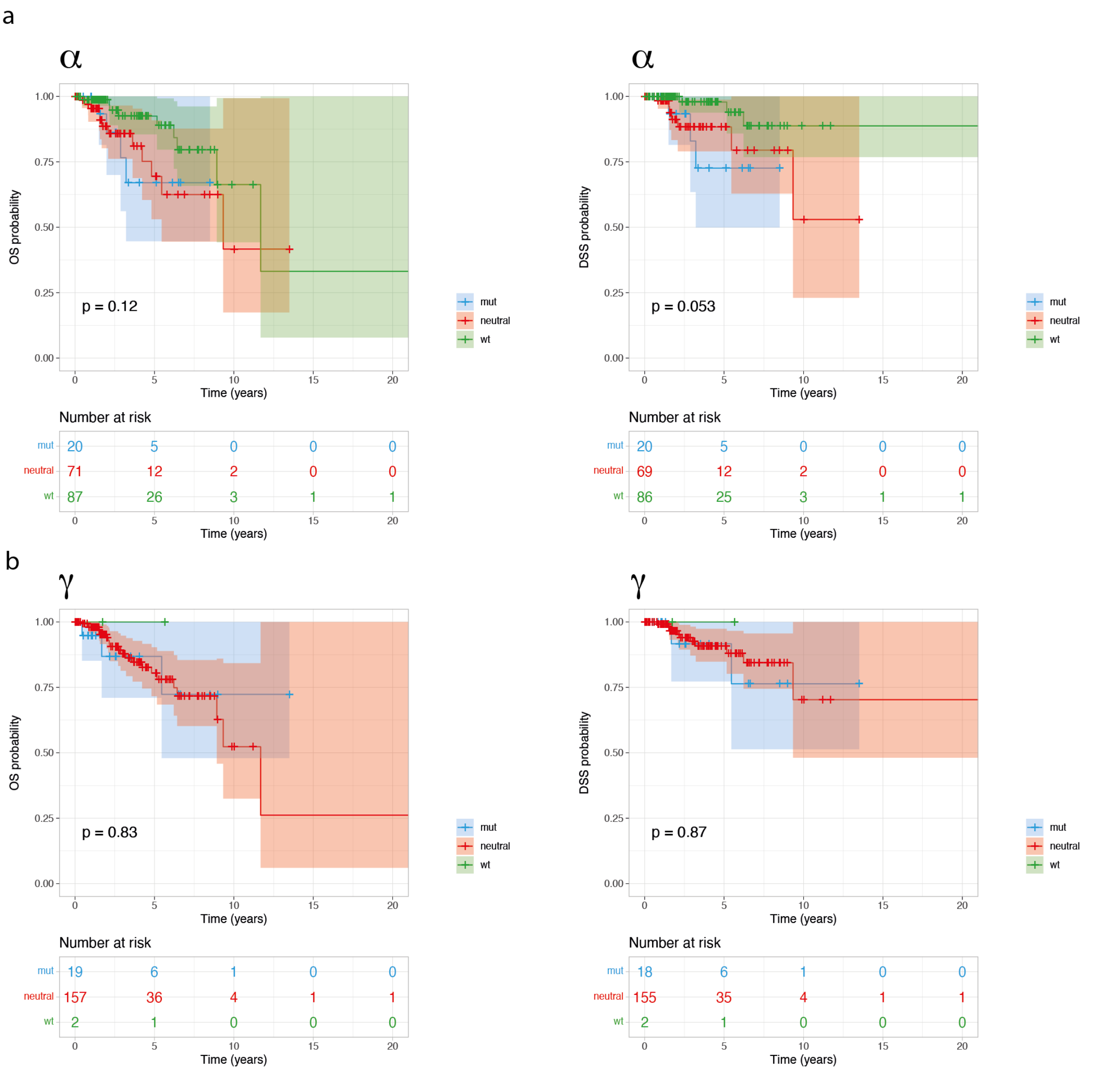


**Supplementary Figure 6 - Survival analysis of mutant allele preferential expression in TCGA tumors. a** – Kaplan–Meier curves of overall and disease-specific survival showing no difference in survival between patients with net differential expression (α) of the *PIK3CA* mutation and those expressing equimolar levels of mutation and wild-type alleles. Disease-specific survival shows a trend towards the worse outcome of those patients whose tumors preferentially express the mutated allele. **b**– Overall and disease-specific survival Kaplan-Meier curves with patients stratified by differential expression driven by cis-regulation (γ), showing no significant difference in survival.
Shown below the graph are the numbers of patients at risk per group throughout time.

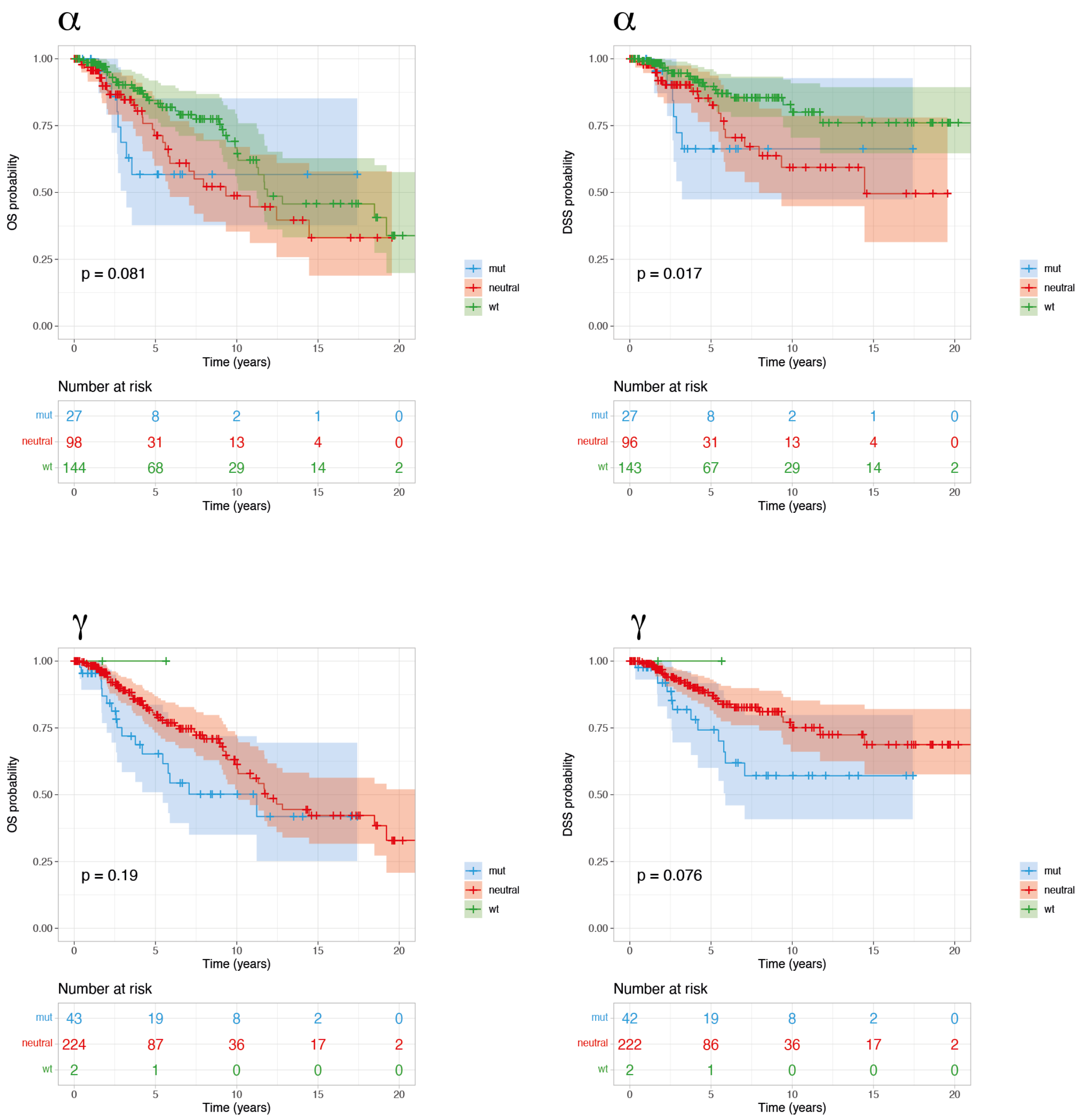


**Supplementary Figure 7 - Survival analysis of mutant allele preferential expression on the two sets (TCGA and METABRIC) of samples combined.** Kaplan–Meier curves of overall and disease-specific survival for patients stratified based on net (α) differential expression of the *PIK3CA* mutation or that due to cis-regulation (γ). Disease-specific survival shows a significant worse outcome of those patients whose tumors display net (α) preferential expression of the mutated allele, a trend also observed when stratification is based on preferential allelic expression due to cis-regulation. Shown below the graph are the numbers of patients at risk per group throughout time.

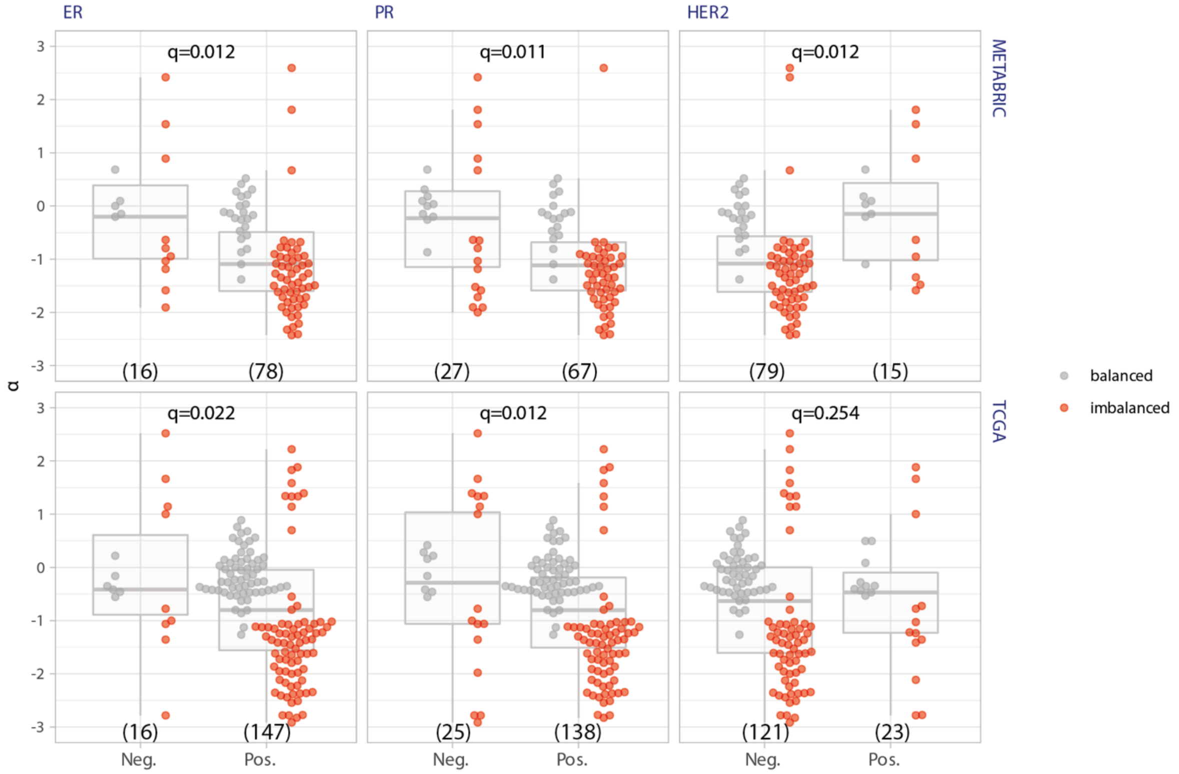


**Supplementary Figure 8- Association analysis between α ratios and ER, PR, and HER2 statuses**. Boxplots for α ratios for the METABRIC (top) and TCGA (bottom) tumors. Each dot represents a sample. In all graphs, samples were colored according to the significance of the allelic expression imbalance and their numbers were indicated in brackets. q-values indicated correspond to the Wilcoxon rank sum test with continuity correction, corrected for multiple testing using the Benjamini & Hochberg method. (ER = estrogen; PR = progesterone; HER2 = human epidermal growth factor 2 receptor).

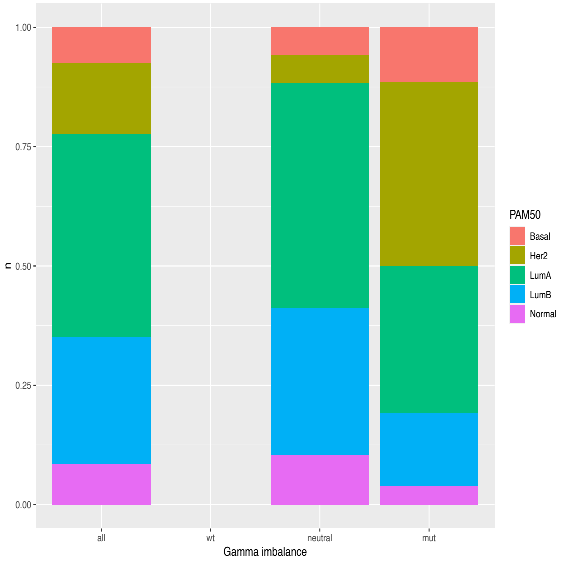


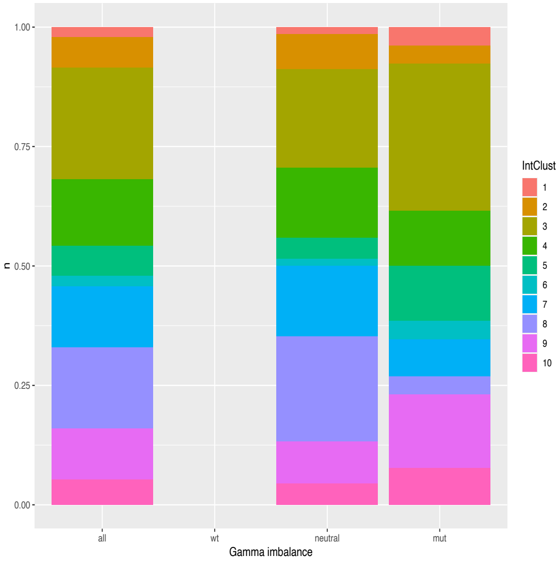


**Supplementary Figure 9 - Distribution (a) PAM50 (Prediction Analysis of Microarray 50) subtypes and (b) IntClust (Integrative Cluster Classification) within samples subdivided in groups according to the significant *PIK3CA's* allelic expression imbalances in the METABRIC set.**

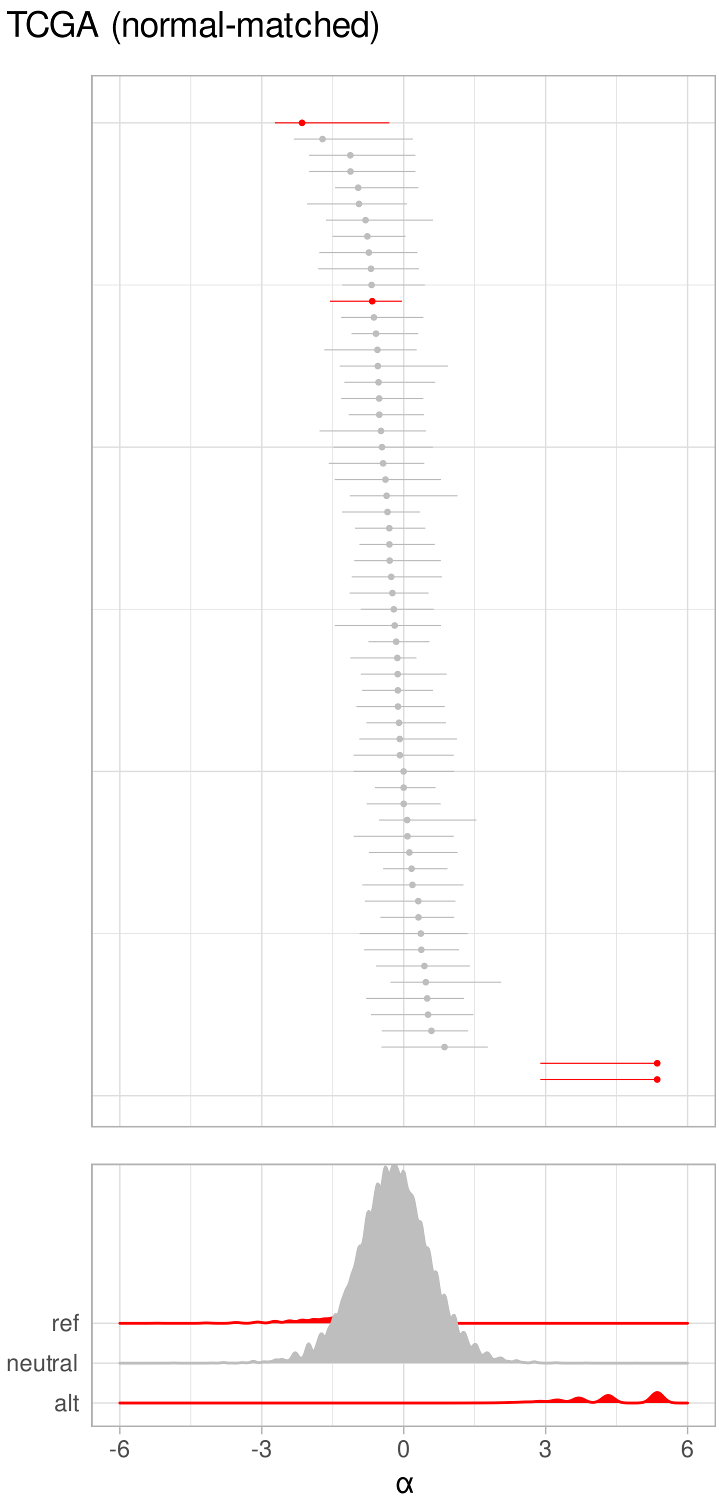


**Supplementary Figure 10 -** Distribution of allelic expression ratios, measured at single-nucleotide polymorphisms located in the *PIK3CA* gene, in normal-matched tissue from patients included in the TCGA study. Samples with significant imbalance are displayed in red.
