# Supplementary material for: Allelic expression imbalance of PIK3CA mutations is frequent in breast cancer and prognostically significant
Source: NPJ Breast Cancer. 2022 Jun 8;8:71. doi: 10.1038/s41523-022-00435-9 (PMC9177727; doi:10.1038/s41523-022-00435-9)
Supplement: Supplementary file 3 — Reporting Summary [file 41523_2022_435_MOESM3_ESM.pdf]

nature portfolio

Corresponding author(s): Ana-Teresa Maia
Last updated by author(s): Nov 13, 2021

Reporting Summary

Nature Portfolio wishes to improve the reproducibility of the work that we publish. This form provides structure for consistency and transparency in reporting. For further information on Nature Portfolio policies, see our Editorial Policies and the Editorial Policy Checklist.

Statistics

For all statistical analyses, confirm that the following items are present in the figure legend, table legend, main text, or Methods section.

n/a	Confirmed	
☐	☒	The exact sample size (*n*) for each experimental group/condition, given as a discrete number and unit of measurement
☐	☒	A statement on whether measurements were taken from distinct samples or whether the same sample was measured repeatedly
☐	☒	The statistical test(s) used AND whether they are one- or two-sided<br>*Only common tests should be described solely by name; describe more complex techniques in the Methods section.*
☐	☒	A description of all covariates tested
☐	☒	A description of any assumptions or corrections, such as tests of normality and adjustment for multiple comparisons
☐	☒	A full description of the statistical parameters including central tendency (e.g. means) or other basic estimates (e.g. regression coefficient) AND variation (e.g. standard deviation) or associated estimates of uncertainty (e.g. confidence intervals)
☐	☒	For null hypothesis testing, the test statistic (e.g. *F*, *t*, *r*) with confidence intervals, effect sizes, degrees of freedom and *P* value noted<br>*Give P values as exact values whenever suitable.*
☐	☒	For Bayesian analysis, information on the choice of priors and Markov chain Monte Carlo settings
☒	☐	For hierarchical and complex designs, identification of the appropriate level for tests and full reporting of outcomes
☐	☒	Estimates of effect sizes (e.g. Cohen's *d*, Pearson's *r*), indicating how they were calculated

Our web collection on statistics for biologists contains articles on many of the points above.

Software and code

Policy information about availability of computer code

Data collection	Data was obtained from the databases where they are deposited, except for the RNAseq data on Metabric samples. This was obtained by capture-based RNA sequencing study (Pereira 2016). Sequencing libraries were prepared using total RNA generated from frozen tissues with a TruSeq mRNA Library Preparation Kit using poly-A-enriched and enriched with the human kinome DNA capture baits. Six libraries were pooled for each capture reaction, with 100 ng of each library and sequenced (paired-end 51bp) on an Illumina HiSeq2000 platform. Sequence data (FASTQ) mapped to reference genome (hg19) were aligned using STAR v2.4.1 (Dobin 2012).
Data analysis	The filtered data and code for the analysis of mutant allele expression imbalances and the survival analysis can be publicly accessed at https://github.com/maialab/npjbcPIK3CA.

For manuscripts utilizing custom algorithms or software that are central to the research but not yet described in published literature, software must be made available to editors and reviewers. We strongly encourage code deposition in a community repository (e.g. GitHub). See the Nature Portfolio guidelines for submitting code & software for further information.

Data

Policy information about availability of data

All manuscripts must include a data availability statement. This statement should provide the following information, where applicable:

- Accession codes, unique identifiers, or web links for publicly available datasets
- A description of any restrictions on data availability
- For clinical datasets or third party data, please ensure that the statement adheres to our policy

Microarray raw data are deposited in the Gene Expression Omnibus under accession number GSE35023. Primary data (BAM files) for DNAseq are deposited at the European Genome-phenome Archive (EGA) under study accession number EGAS00001001753 and may be downloaded upon request and authorization by the

METABRIC Data Access Committee. Primary data (BAM files) for RNAseq are available from the authors upon reasonable request. Primary data (BAM files) for DNAseq and RNAseq from TCGA are deposited in the database of Genotypes and Phenotypes (dbGaP) under the study accession number phs000178.

Field-specific reporting

Please select the one below that is the best fit for your research. If you are not sure, read the appropriate sections before making your selection.

☒ Life sciences ☐ Behavioural & social sciences ☐ Ecological, evolutionary & environmental sciences

For a reference copy of the document with all sections, see nature.com/documents/nr-reporting-summary-flat.pdf

Life sciences study design

All studies must disclose on these points even when the disclosure is negative.

Sample size	Data was used from 64 normal breast samples from healthy women, and from 480 patients from Metabric and 695 patients from TCGA.
Data exclusions	Tumour samples without missense mutations in the gene PIK3CA were excluded.
Replication	We did not perform replication studies, but performed all analysis in the two datasets separately.
Randomization	NA
Blinding	NA

Reporting for specific materials, systems and methods

We require information from authors about some types of materials, experimental systems and methods used in many studies. Here, indicate whether each material, system or method listed is relevant to your study. If you are not sure if a list item applies to your research, read the appropriate section before selecting a response.

Materials & experimental systems

n/a	Involved in the study	
☒	☐	Antibodies
☐	☒	Eukaryotic cell lines
☒	☐	Palaeontology and archaeology
☒	☐	Animals and other organisms
☐	☒	Human research participants
☒	☐	Clinical data
☒	☐	Dual use research of concern

Methods

n/a	Involved in the study	
☒	☐	ChIP-seq
☒	☐	Flow cytometry
☒	☐	MRI-based neuroimaging

Eukaryotic cell lines

Policy information about cell lines

Cell line source(s)	Caldas Lab, University of Cambridge
Authentication	Cell lines have been genotyped and confirmed.
Mycoplasma contamination	Cell lines were routinely tested and were always negative for mycoplasma contamination.
Commonly misidentified lines (See ICLAC register)	NA

Human research participants

Policy information about studies involving human research participants

Population characteristics	The population characteristics have been described in the original publications Maia 2009, Curtis 2012, Wilkerson 2014.
Recruitment	No patients were recruited - secondary analysis of existing data
Ethics oversight	Normal breast and tumor samples from the Metabric project were obtained with the consent from donors and appropriate approval from the Addenbrooke's Hospital Local Research Ethics Committee (REC reference 06/Q0108/221, 07/H0308/161).

TCGA data was accessed in accordance with the Data Use Certification Agreement.

Note that full information on the approval of the study protocol must also be provided in the manuscript.
